# Integrated dataset of anatomical, morphological, and architectural traits for plant species in Madagascar

**DOI:** 10.1016/j.dib.2017.09.004

**Published:** 2017-09-12

**Authors:** Amira Azizan, Emma Guillon, Yves Caraglio, Patrick Langbour, Sébastien Paradis, Pierre Bonnet, Yannick Brohard, Christine Heinz, Nabila Boutahar, Loïc Brancheriau

**Affiliations:** a University of Montpellier, Dept. of Biodiversity, Ecology and Evolution, Montpellier, France; b CIRAD, UR BioWooEB, 34398, Montpellier, France; c CIRAD, UMR AMAP, 34398, Montpellier, France

**Keywords:** Morpho-architectural traits, Plant architecture, Wood anatomy, Madagascar

## Abstract

In this work, we present a dataset, which provides information on the structural diversity of some endemic tropical species in Madagascar. The data were from CIRAD xylotheque (since 1937), and were also collected during various fieldworks (since 1964). The field notes and photographs were provided by French botanists; particularly by Francis Hallé. The dataset covers 250 plant species with anatomical, morphological, and architectural traits indexed from digitized wood slides and fieldwork documents. The digitized wood slides were constituted by the transverse, tangential, and radial sections with three optical magnifications. The main specific anatomical traits can be found within the digitized area. Information on morphological and architectural traits were indexed from digitized field drawings including notes and photographs. The data are hosted in the website ArchiWood (http://archiwood.cirad.fr).

**Specifications Table**

**Table:** 

Subject area	*Botany*
More specific subject area	*Anatomy, morphology and architecture of plant species*
Type of data	*Tables of anatomical traits and morpho-architectural traits*
	*Images of microscope slides for wood anatomy*
	*Field drawings with notes*
	*Photographs*
How data was acquired	*Microscope*
	*Field observations*
Data format	Tables in MSExcel format *.xlsx
	*Images of microscope slides in 24-bit RGB TIFF (1600×1200 pixels)*
	*Digitized field drawings and notes in 24-bit RGB JPG (300 dpi)*
	*Scanned photographs of 24×36* *mm format in 24-bit RGB JPG (600 dpi)*
Experimental factors	–
Experimental features	–
Data source location	*Madagascar*
Data accessibility	*Data package title: ArchiWood dataset*
	*Resource link:*http://archiwood.cirad.fr
	*Identifier: doi*:10.18167/archiwood/1
	*Usage rights: Creative Commons Attribution – NonCommercial – ShareAlike 4.0 International (CC BY-SA-NC 4.0)*

**Value of the data**

• The dataset consolidates anatomical, morphological, and architectural traits of plant species from different sources (xylotheque, field notes, and photographs).
• The described traits of tropical plant species can be useful to understand the biogeographical variation within species and genera in plant anatomy regarding the ontogeny and structure of sampled plants.
• To understand the diversity of wood characteristics and technological behaviors that directly governed the choice of tropical timber use.
• To understand the relationship between tropical wood structure and certain physical, mechanical, chemical, and biological properties of the material.

## 1 Data

Madagascar is an important insular hotspot for biodiversity conservation [11]. More than 80% of the currently known flora species are endemic to the island [4]. Endemic tropical species in Madagascar are well known for its important value in ecology and economy but are predicted to face mass extinction in the near future because of global warming and deforestation [2], [3]. Identification of Malagasy vascular plant species was documented and can be accessed via the website Tropicos (Madagascar catalogue, http://www.tropicos.org/Project/Madagascar).

The architectural analysis on tropical species as described by [1], [7], [8] emphasize on the dynamics of growth and structure of a plant species in the competitive nature of the forest, thereby conforming to its architectural model. Recent studies of plant architecture may not only provide complimentary information for species-level identification purposes, but also for understanding plant structure evolution within a clade [10] and their ecological strategies [5], [6]. Together with stem anatomy, architectural variations involving the branching process, rhythmicity, and orientation can be comparatively analyzed to study the evolution of growth in both temporal and spatial contexts [9]. This approach can also provide guidelines for tree selection and management in agroforestry practices [12].

The dataset gathers anatomical, morphological, and architectural traits of endemic plant species. The aims is to promote and to make more accessible for scientific and public use a specific part of the CIRAD wood collection and unpublished morpho-architectural data from Madagascar. More than 1000 microscopic anatomical wood slides, exclusively available in this open access dataset, were sampled since the early 20th century and compiled with more than 500 digitized field notes such as botanical illustrations and photographs. Because diagrammatic representations of morpho-architectural traits are essential to assess plant structure and growth, digitized illustrations provided here may serve as a reference for future architectural analysis and application. Metadata from approximately 250 indexed plant species also provide new insights to species identification and scientific research, such as in functional ecology or systematics as well as for forest conservation management.

## 2 Experimental design, materials and methods

### 2.1 Microscopic structure of wood

**Fig. 1:**
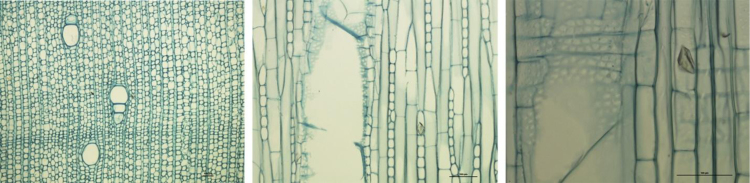
Digitized wood slides highlighting anatomical traits. From left to right: transverse (×40), tangential (×100) and radial (×200) sections of *Givotia madagascariensis* Baill.

For each species, three microscope slides were prepared consisting of transverse, tangential, and radial sections (Fig. 1). The slides were then digitized using a camera Olympus DP71 mounted onto a microscope Olympus BX60. Each slide was digitized with three optical magnifications: ×40, ×100, and×200. The images (1600×1200 pixels) were then processed by Archimed Microvision® software and stored in 24-bit RGB TIFF. Within each image, the main specific anatomical traits can be found, and these traits were indexed following the List of Microscopic Features for Hardwood Identification from the IAWA (http://iawa-website.org/).

### 2.2 Morphology and architecture of plant structure

**Fig. 2:**
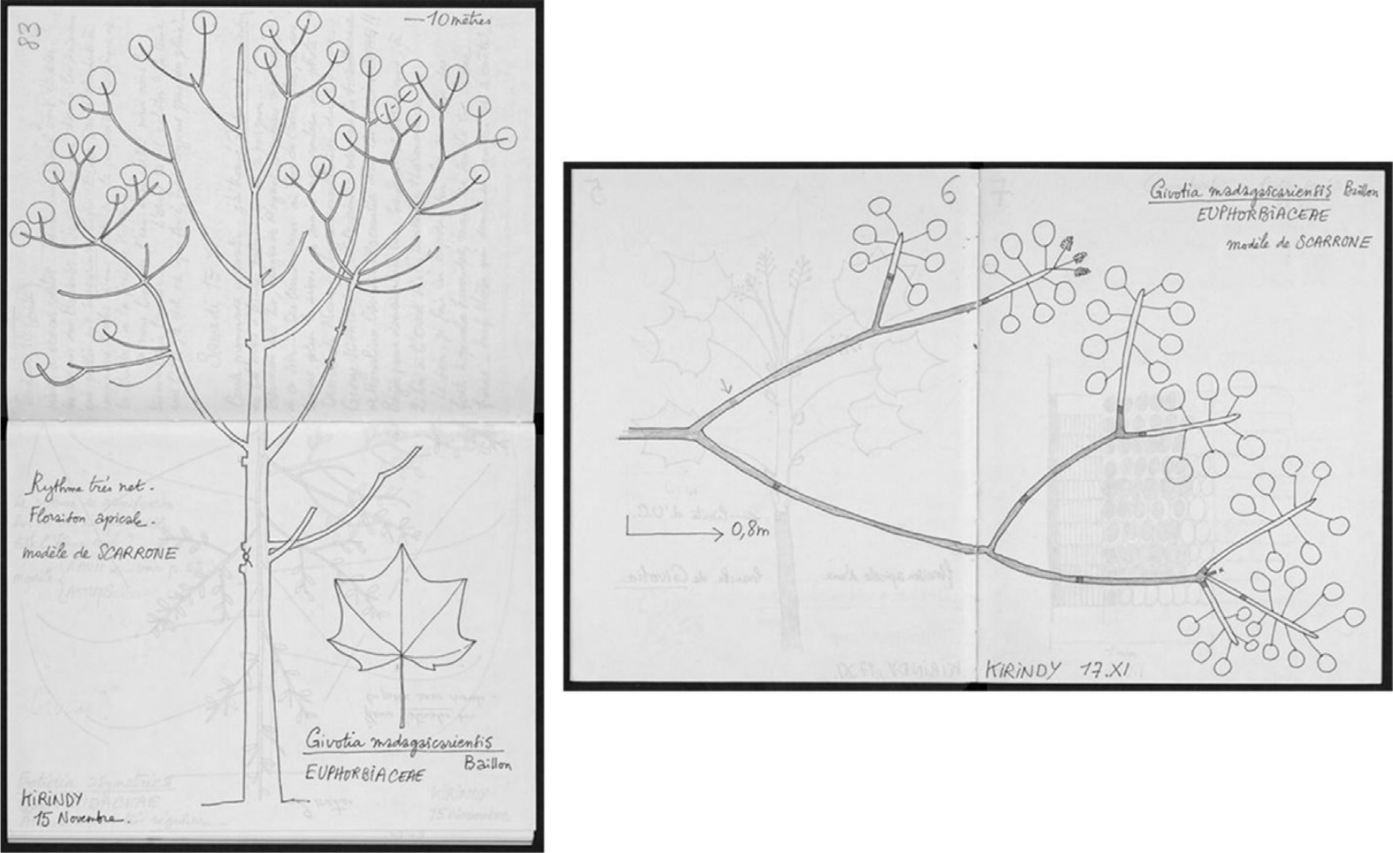
Fieldwork illustrations of morpho-architectural traits. Diagrammatic representation of *Givotia madagascariensis* Baill for whole plant (left) and branching system (right).

Information on morphological and architectural traits were indexed from illustrations of digitized field notes (300 dpi 24-bit RGB JPEG) (Fig. 2) and scanned photographs of 24×36 mm format (600 dpi, 24-bit RGB JPEG) from various researches in Madagascar, for extended visual information. The traits were indexed following the modalities as reviewed in [1], [7], [8]. Both anatomical and morphological traits may not apply to all of the species available in the dataset.

### 2.3 Temporal coverage

Concerning the wood collection, the anatomical slides were sampled since 1937. The field notes were reported since 1964 for the photographs, and since 1970 for the illustrations. Each document was digitized and indexed between June 2015 and the end of 2016. This information were never published.

### 2.4 Taxonomic coverage

The dataset gathered information on 102 families and 244 genera of angiosperm species (with the addition of a bryophyte). For these species, the updated taxonomic names were provided and retrieved from The Plant List (http://www.theplantlist.org/). Some traits were specified at the family or genus level.

## Funding

This work was supported by the French Digital Scientific Library (BSN5, 2014, ArchiWood project).
